# A Double Transducer for High Precision Ultrasonic Time-Domain Reflectometry Measurements

**DOI:** 10.3390/s150715090

**Published:** 2015-06-26

**Authors:** Sam Stade, Tuomas Hakkarainen, Mari Kallioinen, Mika Mänttäri, Tuure Tuuva

**Affiliations:** 1 Laboratory of Separation Technology, School of Engineering Science, Lappeenranta University of Technology, P.O. Box 20, Lappeenranta FI-53851, Finland; E-Mails: mari.kallioinen@lut.fi (M.K.); mika.manttari@lut.fi (M.M.); 2 Laboratory of Physics, School of Engineering Science, Lappeenranta University of Technology, P.O. Box 20, Lappeenranta FIN-53851, Finland; E-Mails: tuomas.t.hakkarainen@lut.fi (T.H.); tuure.tuuva@lut.fi (T.T.)

**Keywords:** ultrasonic time-domain reflectometry, UTDR, time-of-flight measurement, environmental compensation, reference transducer, double transducer, piezoelectric

## Abstract

Membrane fouling, where unwanted particles accumulate on the membrane surface and reduce its permeability, causes problems in membrane filtration processes. With ultrasonic time-domain reflectometry (UTDR) it is possible to measure the extent of membrane fouling and hence take actions to minimize it. However, the usability of UTDR is very limited to constant filtration conditions if the sonic velocity, which has a great impact on UTDR measurement accuracy, is unknown. With a reference transducer the actual sonic velocity can be measured. This requires another transducer to be installed in the module, where there may be only limited space or the module dimensions may not be suitable for the reference transducer. A double transducer described in this study eliminates the need for a separate reference transducer because in the double transducer the reference measurement is included in the design of the transducer holder. Two sensors in the same holder require less space. Other advantage is that the double transducer can be placed near the measurement target and hence the local sonic velocity can be determined.

## 1. Introduction

One of the main problems in membrane filtration is fouling. Fouling can be described as an accumulation of unwanted material on the membrane surface hence decreasing its permeability. To better understand this phenomenon, real-time monitoring is needed. The data can then be used to choose the best possible filtration conditions, material choices, cleaning procedures or pre-treatment to minimize fouling. Membrane applications may have different fouling mechanics due to different types of foulants, thereby different filtration processes each require their own monitoring setup which cannot be too expensive and it has to give the necessary data about the process. The data can be a quantitative measurement or qualitative information about fouling layer thickness to alert a user about the changes occurring on the membrane surface due to the fouling.

Ultrasonic time-domain reflectometry (UTDR) is a non-invasive real-time monitoring method which has been used to monitor membrane compaction [1,2,3,4], fouling [5,6,7,8,9], cleaning [10,11] and casting processes [12]. It is based on a time-of-flight measurement; the distance to the membrane surface is determined from the time it takes for an ultrasonic pulse to travel from a transducer to the membrane surface and back to the transducer. Theoretical accuracy of 0.75 µm can be achieved if time is measured with 1 ns accuracy [3]. However, other error sources, for instance non-constant sonic velocity, may greatly affect this accuracy. Despite the accuracy limitations, UTDR has been reported to be one of the few promising technologies that could be applied to commercial-scale filtration modules [13].

Sonic velocity is often assumed to be constant in UTDR measurements. This limits the use of UTDR measurements to applications where the operation conditions which affect the sonic velocity are constant as well. Recently Stade *et al.* [14] published a method for increasing UTDR measurement possibilities and accuracy with a reference transducer. A reference transducer is used to measure a known distance in the process, for example the membrane filtration module channel width, which allows determination of the sonic velocity of the fluid inside the module. When the sonic velocity is measured the process operation conditions can vary and UTDR can still be used. Stade *et al.* [14] have demonstrated earlier in three different experiments that the measurement accuracy in practice varied between 0.6–2.0 µm when the temperature was changed from 25 to 60 °C, pressure varied from 0.1 to 0.5 MPa and the measurement distance was 17.8 mm.

**Figure 1:**
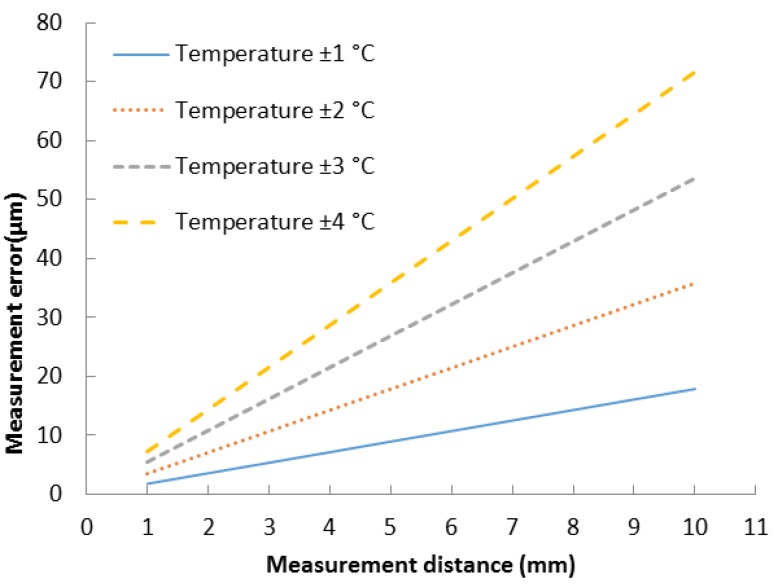
Maximum measurement errors caused by changes in the sonic velocity due to temperature changes. Calculated from an empirical model made by Belogol’skii *et al.* [16] Temperature = 25 °C.

The measurement setup may greatly affect the measurement accuracy in practice [15]. The module has to be robust so that pressure or temperature changes does not affect its dimensions. High precision UTDR measurements with a reference transducer favor short distances where the ultrasonic path of the distance measurement is as representative as possible with the path of the reference transducer measurement. Even small differences in sonic velocities in those paths may cause an error when the operation conditions vary. For example a 1 °C temperature change for 25 °C water, when the measurement distance is 17.8 mm, leads to a 32 μm error. If the measurement distance were to be as short as 5 mm the error would be ~9 μm. The same error is valid for UTDR measurements where the sonic velocity is assumed to be constant. Figure 1 shows how the measurement distance and temperature change of water affect the measurement error.

A new transducer design is presented in this paper. The idea was developed from our earlier paper where the reference measurements to determine the sonic velocity were made with the aid of an additional transducer [14]. As installation of the separate reference transducer may be problematic in narrow filtration channel modules, a double transducer was developed. It has two piezoelectric sensors built together in one transducer holder. The sensors are at different distances which allows determination of the local sonic velocity through the measurements. This enables measurements in places where is not enough space for a separate reference transducer.

## 2. Experimental Section

**Figure 2:**
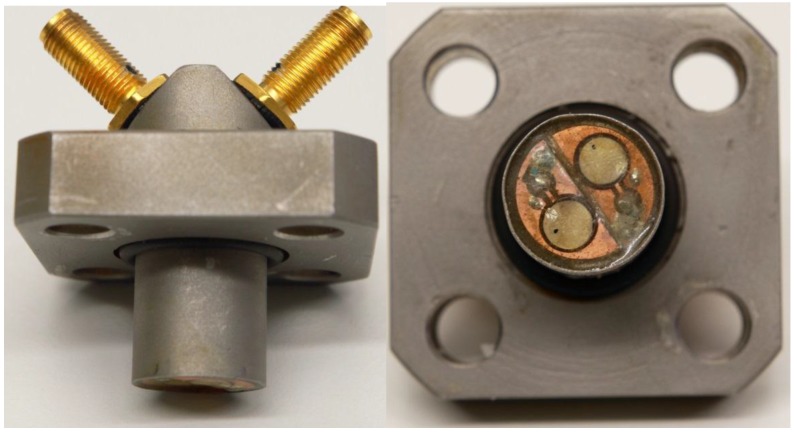
Photographs of the double transducer.

An ultrasonic transducer has a sensor which is a piezoelectric crystal. It can send ultrasonic waves and detect them electrically. The double transducer is a device where two crystals are together (Figure 2). Crystals are made out of lead zirconate titanate (Ferroperm, Humlebaek, Denmark) and they are designed for underwater applications [17]. The commercial name of these crystals is Pz26 (Navy I). The advantages with these crystals are their small size, approximately 10 MHz resonance frequency and relatively high coupling factor compared with other crystals in this class. Resistors, crystals and the tip of holder are covered with an epoxy to keep the acoustic impedance applicable between water and crystals and to protect the electronics from the solution.

The holder is made out of stainless steel to withstand the pressure when used in membrane modules. It has O-ring insulation between holder tip and SubMiniature version A (SMA) connectors. The holder has four 10 mm bolt attachment points to secure robust attachment to the adjustable arm or the membrane module.

The holder has two coaxial SMA 50 Ω input connectors on the top where signals go through leads to the tip of the holder. At the tip of the holder there are one resistor and sensor for both inputs and they are coupled electrically in parallel. Both inputs are also coupled electrically identically. The other end is coupled to a signal lead and grounded to the holder via the SMA connector frames and shielded coaxial cables were used to reduce the differential mode noise. Resistors were coupled electrically in parallel to the crystals to decrease the resonance damping time. This will decrease the amplitude of the pulse as it increases the energy losses in the transmission line but it will also settle the crystal element quicker in order to be damped fast enough to receive echo pulses from close distances.

**Figure 3:**
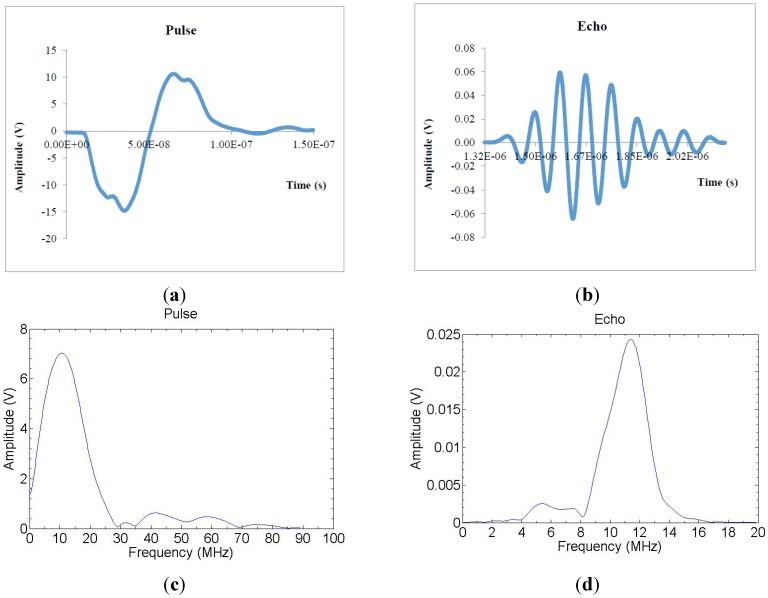
(**a**,**b**) The time-domain responses of the sent pulse and received echo; (**c**,**d**) Frequency-domain responses.

**Figure 4:**
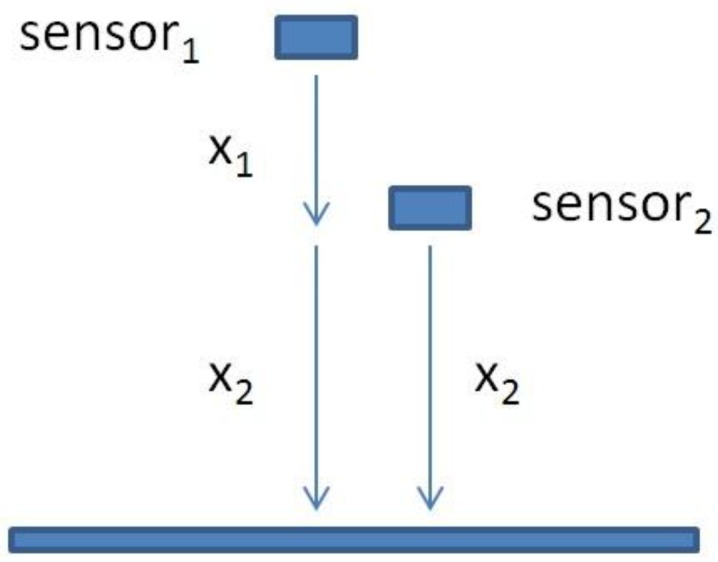
The idea behind the double transducer is the fixed distance between sensors. The fixed distance can be used to determine the local sonic velocity which improves the measurement accuracy if the sonic velocity is not constant. X_1_ is the fixed distance and X_2_ is the distance to the measured surface.

**Figure 5:**
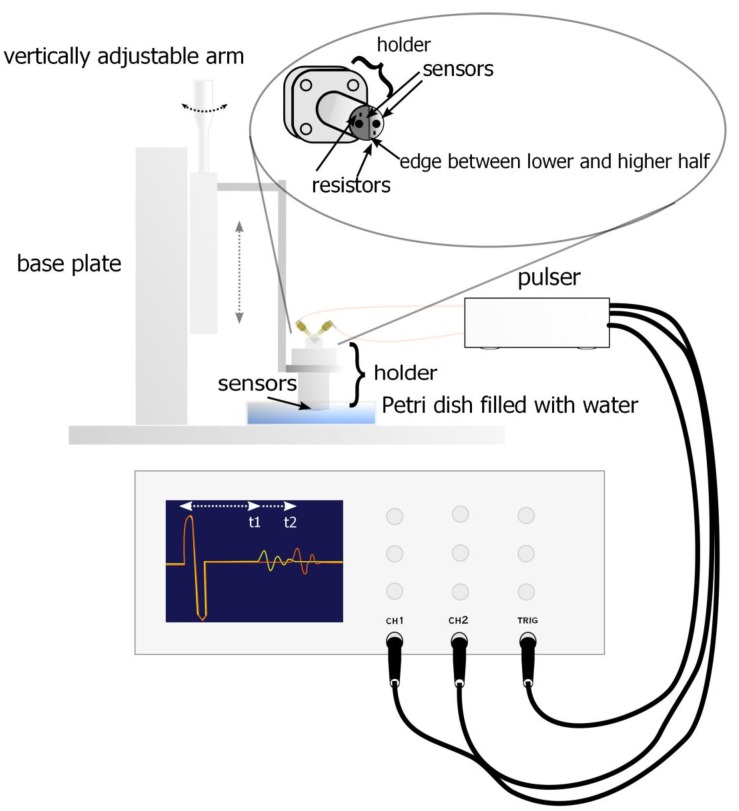
Experimental setup for UTDR. Ultrasonic transducer is attached to an adjustable holder which distance can be changed with a micrometer screw. Ultrasonic pulse is generated with a pulser and monitored with an oscilloscope.

An electric pulse was generated by a pulser which sends 10 MHz electric pulses to both crystals at the same time. The pulser device has five terminals; two outputs for the transmission signals to the crystals, two outputs for the two oscilloscope channels and one output for the synchronization signal. The pulse is triggered in the oscilloscope with an external trigger signal which is timed so that the signal is received from the start where the pulse is generated and the trigger signal ends at the time when the signal is assumed to complete its roundtrip from the sensor to the bottom of the Petri dish and back to the sensor. The time difference between the sent and received pulses (echoes) was detected with an oscilloscope. An example of the sent pulse and received echo in time- and frequency domain are shown in the Figure 3a–d. The fundamental idea of the double transducer is to measure the sonic velocity simultaneously with the distance measurement under variable environmental conditions such as temperature changes. The fixed distance between sensors of the double transducer (Figure 4) can be used to calculate the sonic velocity from the time difference between these two signals. The fixed distance has to be determined for the double transducer. It can be done with a simple “calibration” measurement using a device like the one shown in Figure 5.

In our experimental calibration setup, the double transducer was attached to an adjustable transducer holder (Figure 5). The tip of the double transducer was held in a Petri dish filled with water. The calibration measurements were done by adjusting the micrometer adjustable arm with constant length steps. After each step, the time differences between the sent and received pulses were recorded from both sensors.

The temperature was held constant (23 °C) during the calibration. Acquired data set is a function of time related to distance from both sensors and they form two linear lines. Each linear equation consist a slope which is the sonic velocity and a constant part which is the distance between the sensor and the sample. The fixed distance is calculated from the distance difference between each sensor and the sample.

The calibrated double transducer was tested in fouling experiments. A calcium carbonate fouling layer was created in the dead-end filtration from undersaturated NaHCO_3_ and CaCl_2_ solutions. Concentration of Ca^2+^ and HCO_3_^−^ was 1 g/L, temperature was held 30 °C and filtration pressure was 1 MPa. Desal 5 DK membrane was used in the experiment. After precompaction of the membrane, CaCl_2_ solution was added in the feed tank and pH was adjusted so that the solution was undersaturated according to Langelier saturation index. After that the NaHCO_3_ solution was added to the feed tank. As the filtration started, the concentration near the membrane surface increased due the semi-permeable membrane and the undersaturated solution turned saturated and calcium carbonate crystals formed the fouling layer. Permeate was returned to the feed tank. Permeate fluxes and the fouling layer thicknesses were measured. After the experiment, the membrane fouling layer was analyzed with the scanning electron microscope (SEM).

## 3. Results and Discussion

**Figure 6:**
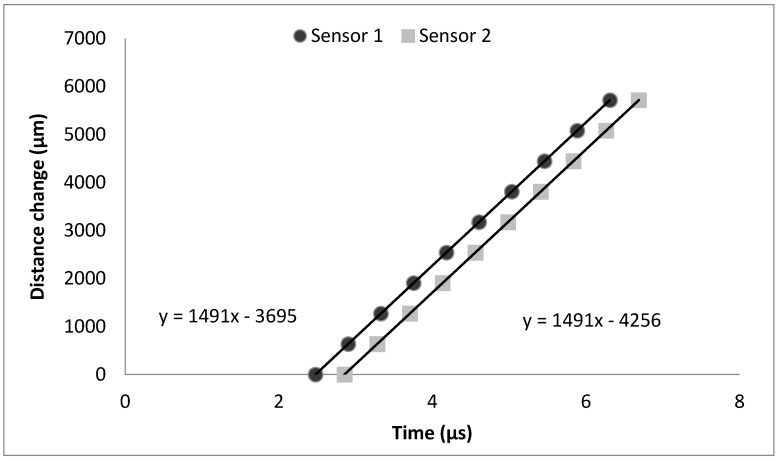
Calibration measurement of double transducer. Time was measured at 10 different distances. Sonic velocity was same with both transducers (1491 m/s) and distance between transducers is 561 µm (= 4256 − 3695).

The calibration results are shown in Figure 6. As can be seen from the figure, the measured time *versus* distance change forms two linear lines. The sonic velocity is determined from the slopes of these lines and it correlates with the value calculated with an equation developed by Belogol’skii, Sekoyan *et al.* [16] (1491 m/s, 1 bar, 23 °C). The equation can be used to calculate the sonic velocity in water as a function of temperature and pressure. The equation and its use have been previously explained in depth by Stade *et al.* [14].

The fixed distance between the sensors is calculated from the constant values of the linear regressions. The constant value gives information about how far the measuring sensor is from the target surface. The difference between these two values is the fixed distance between them (561 μm). After the calibration, the transducer can be deployed inside the membrane module, or any other apparatus where the time-domain distance measurements are needed, and this fixed distance can be exploited for sonic velocity calculation. When measuring, the time difference between these two sensors can be used to determine the sonic velocity as shown in the Equation (1):

(1) $$C=\frac{2s_{f}}{∆t}$$

where *C* is the sonic velocity in the media, *s_f_* is the fixed distance between the transducers, and *Δt* is the measured time difference between sensors 1 and 2. Now the determined sonic velocity *C* can be used to calculate the distance to the target surface with Equation (2):

(2) $$∆s_{1}=\frac{Ct}{2}$$

where *Δs*_1_ is the distance between sensor 1 and the target surface and *t* is the measured time from the sensor 1. The accuracy of the double transducer is better when the transducer is closer to the target surface. This is due the relatively short fixed distance used to determine the sonic velocity. If the target surface is further away than the fixed distance, the accuracy will decrease as a multiple of the distance.

For example in the older reference transducer setup presented by Stade *et al.* [14], a separate reference transducer worked better when the measurement distance was 17,800 μm and the fixed distance of the double transducer shown in this paper is only 561 μm. However, the filtration channels in many membrane modules are only a few millimetres high and there is not enough space for a separate reference transducer. In those cases, where the measurements can be done from the distances close to the fixed distance, this kind of double transducer design is ideal.

As a requirement for use of the double transducer, it has to be integrated inside the module where it can be immersed in the fluid. Without the contact to the fluid, the sonic velocity obviously cannot be determined. This will rule out the use of the double transducers as “through the top plate” devices which has been a very common way to measure time- and amplitude-domain in membrane applications.

One of the benefits of the integrated sensors is better sensitivity as the signal is not attenuated and scattered by the top plate. As a downside, the integrated sensors’ lifetime may be reduced by the filtration solution. The double transducer has the other sensor at a different distance thereby there is a hollow point which may collect impurities and affect the fluid flow properties inside the module. Also when the measurements are done from close distances (<2 mm) damping of the crystal may be a problem. The crystal may still be vibrating when the echo from the measured surface is returning. Of course this kind of problem can be solved with appropriate software or electronics which record the “background” signal and remove it from the measurement signal. However, this is the only solution currently available for the UTDR measurements in changing filtration conditions where there only is space for one transducer.

**Figure 7:**
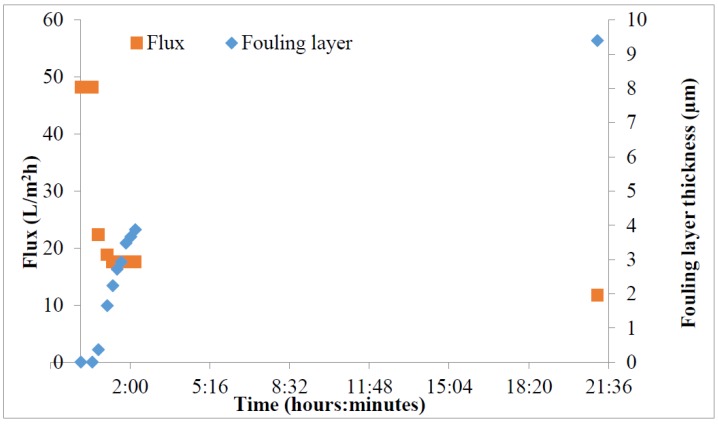
Results of the fouling experiment. The flux and the fouling layer thickness were measured during the filtration.

**Figure 8:**
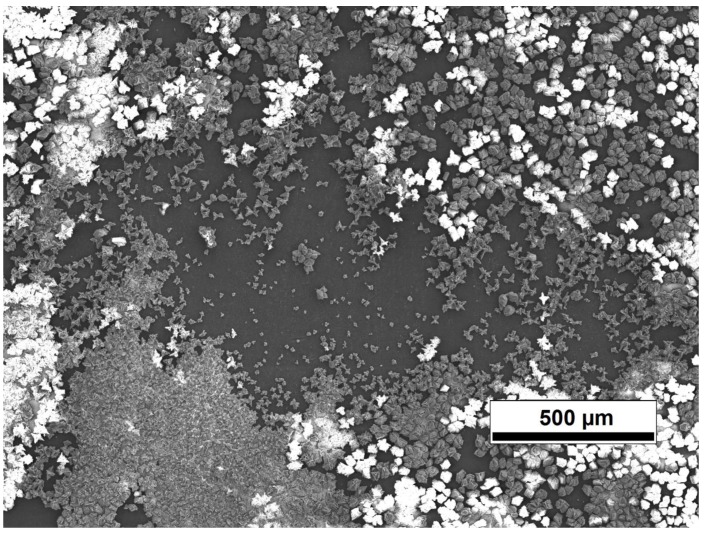
The SEM image of the calcium carbonate fouling on the Desal 5 DK membrane surface.

The fouling test results are shown in the Figure 7 and Figure 8. As can be seen from the Figure 7, the fouling layer started to grow when the filtration started and the flux started to decrease. The filtration continued over the night and next day the filtration was ended and the membrane was analysed with the SEM. The fouling layer can be clearly seen from the SEM image (Figure 8).

The fouling layer thickness measured with the UTDR was 9.4 µm in the end of the experiment. However, the calcium carbonate crystals varied from 5–35 µm in the SEM image. The UTDR may not be able to detect the largest single crystals; it measures an average distance of the sonic beam area that is reflected back and is efficient enough to be detected with the sensor. Surface roughness and sharp shapes of the particles may scatter the signal. However, the scattering should not be the problem in this case as the diameter of the sensor element is 5 mm and the distance is 1 mm. Also the area of coverage of the fouling may affect the distance measurement as there were almost clean, non-fouled, areas on the membrane surface.

The sonic velocity decreased during the filtration. In the beginning of the experiment the sonic velocity was 1690 m/s and it decreased as the fouling layer increased to 1666 m/s. The concentration of the solutes decreases due the crystallization of the calcium carbonate on the membrane surface and it has impact on the sonic velocity. As a down side of the experiment, it was performed in the dead-end filtration mode and it may affect the determination of the sonic velocity with the double transducer. The double transducer detects the sonic velocity near the transducer and the sonic velocity may be different near the membrane surface. However, the measured sonic velocity near the transducer is still better than the estimated constant value for the sonic velocity as the UTDR have been used before.

## 4. Conclusions

A novel transducer design for the UTDR technique has been presented in this manuscript. This so called “double transducer” has two integrated sensors in one transducer and the sensors have a fixed distance between them. This fixed distance can be used to determine sonic velocities in the UTDR measurements which are affected by the filtration conditions and thus increase its accuracy and possibilities. The design of the double transducer enables integration of the transducer close to the target surface and inside narrow (<5 mm high) filtration channels.

Without environmental compensation (a reference transducer) the UTDR is very limited to constant conditions and even small changes in temperature may lead to a notable measurement error, for example a 1 °C temperature change of water, when the measurement distance is 2 mm will lead to a 3.6 μm error. Even as small an error as 3.6 μm is notable when the UTDR is used as an early-stage fouling monitoring.

## References

[1] Stade S., Kallioinen M., Mikkola A., Tuuva T., Mänttäri M. (2013). Reversible and irreversible compaction of ultrafiltration membranes. Sep. Purif. Technol..

[2] Aerts P., Greenberg A.R., Leysen R., Krantz W.B., Reinsch V.E., Jacobs P.A. (2001). The influence of filler concentration on the compaction and filtration properties of Zirfon®-composite ultrafiltration membranes. Sep. Purif. Technol..

[3] Peterson R.A., Greenberg A.R., Bond L.J., Krantz W.B. (1998). Use of ultrasonic TDR for real-time noninvasive measurement of compressive strain during membrane compaction. Desalination.

[4] Reinsch V.E., Greenberg A.R., Kelley S.S., Peterson R., Bond L.J. (2000). A new technique for the simultaneous, real-time measurement of membrane compaction and performance during exposure to high-pressure gas. J. Membr. Sci..

[5] Mairal A.P., Greenberg A.R., Krantz W.B., Bond L.J. (1999). Real-time measurement of inorganic fouling of RO desalination membranes using ultrasonic time-domain reflectometry. J. Membr. Sci..

[6] Li J., Sanderson R.D., Hallbauer D.K., Hallbauer-Zadorozhnaya V.Y. (2002). Measurement and modelling of organic fouling deposition in ultrafiltration by ultrasonic transfer signals and reflections. Desalination.

[7] Li J., Sanderson R.D., Jacobs E.P. (2002). Non-invasive visualization of the fouling of microfiltration membranes by ultrasonic time-domain reflectometry. J. Membr. Sci..

[8] Li J., Sanderson R.D., Chai G.Y. (2006). A focused ultrasonic sensor for *in situ* detection of protein fouling on tubular ultrafiltration membranes. Sens. Actuators B Chem..

[9] Sim S.T.V., Chong T.H., Krantz W.B., Fane A.G. (2012). Monitoring of colloidal fouling and its associated metastability using Ultrasonic Time Domain Reflectometry. J. Membr. Sci..

[10] Mairal A.P., Greenberg A.R., Kratz W.B. (2000). Investigation of membrane fouling and cleaning using ultrasonic time-domain reflectometry. Desalination.

[11] Li J., Hallbauer D.K., Sanderson R.D. (2003). Direct monitoring of membrane fouling and cleaning during ultrafiltration using a non-invasive ultrasonic technique. J. Membr. Sci..

[12] Kools W.F.C., Konagurthu S., Greenberg A.R., Bond L.J., Krantz W.B., van den Boomgaard T., Strathmann H. (1998). Use of ultrasonic time-domain reflectometry for real-time measurement of thickness changes during evaporative casting of polymeric films. J. Appl. Polym. Sci..

[13] Chen V., Li H., Fane A.G. (2004). Non-invasice observation of synthetic membrane processes–a review of methods. J. Membr. Sci..

[14] Stade S., Kallioinen M., Mänttäri M., Tuuva T. (2014). High Precision UTDR Measurements by Sonic Velocity Compensation with Reference Transducer. Sensors.

[15] Karjalainen A. (2010). Online Ultrasound Measurements of Membrane Compaction. Ph.D. Thesis.

[16] Belogol’skii V.A., Sekoyan S.S., Samorukova L.M., Stefanov S.R., Levtsov V.I. (1999). Pressure dependence of the sound velocity in distilled water. Meas. Tech..

[17] Piezoceramic Components Production. http://www.meggittsensingsystems.com/capabilities/piezoceramic-components-production.html.

